# Hepatitis B virus variants in an HIV-HBV co-infected patient at different periods of antiretroviral treatment with and without lamivudine

**DOI:** 10.1186/1471-2334-4-29

**Published:** 2004-08-31

**Authors:** Eneida A Santos, Michel VF Sucupira, Juçara Arabe, Selma A Gomes

**Affiliations:** 1 Department of Virology, Oswaldo Cruz Institute, Rio de Janeiro, RJ 21045-900, Brazil; 2 Gaffrée and Guinle University Hospital, Rio de Janeiro, RJ 20270-004, Brazil

## Abstract

**Background:**

Lamivudine inhibits replication of both human immunodeficiency virus (HIV) and hepatitis B virus (HBV) and is commonly used as part of antiretroviral therapy. The main limitation in the use of lamivudine is resistant mutation selection. Most of these mutations affect the YMDD motif of the HBV DNA polymerase. The resistance occurs through M550V or M550I aminoacid replacements. The M550V variation may be accompanied by L526M mutation, notably in HIV-HBV co-infected patients. The aim of this study was to investigate mutations associated with lamivudine resistance in a hemodialysis patient chronically co-infected with HIV-1 and HBV, who was submitted to several antiretroviral treatments.

**Methods:**

HBV isolates derived from three blood samples collected at different times of antiretroviral therapies with and without lamivudine, were titred and submitted to nucleotide sequencing.

**Results:**

HBV isolate derived from a sample collected in 1999 during an antiretroviral treatment with lamivudine showed the lamivudine resistant double mutation (L526M, M550V). However, no mutation associated with lamivudine resistance was observed in the HBV genome derived from the sample collected during a period of treatment without lamivudine (2001). After reinstitution of lamivudine (2002), the predominant HBV population exhibited a rare triple mutation (V519L, L526M, M550V), which has previously been associated with an *in vitro *reduction of virus antigenicity (escape mutant). HBV DNA was detected at high levels (10^8^–10^9 ^copies/ml) in the three blood samples.

**Conclusions:**

Reintroduction of lamivudine as part of antiretroviral treatment in a patient who had developed lamivudine resistant HBV strains favored the predominance of an HBV isolate with reduced antigenicity. The absence of hepatitis acute exacerbation in this patient may be correlated to the absence of significant variations of the viral load, which was independent of the presence of mutations in the HBV DNA polymerase.

## Background

Hepatitis B virus (HBV) and human immunodeficiency virus (HIV) share common routes of transmission, mainly sexual, parenteral and vertical. Therefore, the prevalence of HBV serological markers is higher among HIV infected patients than in non-HIV infected individuals [1,2]. Considerable variations in the prevalence of HBV markers have been observed in HIV patients according to the geographical region and risk of exposure [3]. Recently, a prevalence of 68% of antibodies against hepatitis B core antigen (anti-HBc) was observed in HIV infected patients living in Rio de Janeiro, Brazil [4]. Co-infection with HIV interferes with the natural history of HBV infection and is associated with higher HBV DNA levels [5,6]. A more common progression to cirrhosis, despite a milder histological necro-inflammatory activity, has also been observed in cases of HIV-HBV co-infection [5].

Lamivudine is a nucleoside analogue that inhibits the reverse transcriptase activity of both HIV and HBV [7] and is commonly used in the treatment of both viral infections [8,9]. The HBV DNA loss due to lamivudine treatment is usually accompanied by significant histological and biochemical improvement [10]. The major limitation in the use of lamivudine is the selection of resistant mutations that may arise and accumulate during therapy. Most of these mutations usually affect the YMDD motif of the HBV DNA polymerase, by replacement of a methionine residue at position 550 with either valine (M550V) or isoleucine (M550I). Such mutations have notably been reported in HIV-HBV co-infected patients [11,12] who develop lamivudine resistance at an annual rate of 20%, with a projected rate of 90% after four years of therapy [13].

The consequences of drug resistance mutations for the evolution of HBV induced liver disease are currently under study. Hepatitis B acute exacerbation has been described after withdrawal of lamivudine therapy [14]. Such an exacerbation has also been associated with the appearance of YMDD mutants [14,15] and with a rapid increase of viral load [16].

The present study was performed to characterize HBV variants and genetic patterns of lamivudine resistant HBV strains in a patient co-infected with HIV-1 at different periods of an antiretroviral treatment with and without lamivudine.

## Methods

### Patient characteristics and serological markers

A 30-year-old male patient initiated a hemodialysis treatment in August 1995. At that time, he was negative for hepatitis B surface antigen (HBsAg) as well as for anti-HBc and anti-HIV antibodies. The patient received three blood transfusions during the first trimester of 1996 and developed a posttransfusional hepatitis. He became HBsAg and anti-HIV positive and remained positive for both markers in all routine tests performed between 1996 and 2002. Data of other tests, such as serum transaminases, CD4 levels, and HIV load, performed in external laboratories between 1995 and 2002, were available. Resistance to different HIV therapies was evaluated by the medical staff based on HIV load and CD4 levels. HIV treatment started in April 1996, with a combination of zidovudine (AZT) and didanosine (ddI). In November 1996, this treatment was modified to a combination of AZT and lamivudine which was maintained until March 1997, when treatment was once again changed to triple therapy with AZT, lamivudine and invirase. In May 1999, high HIV load (3.1 × 10^5 ^copies/ml) was observed, indicating HIV resistance. At that time the patient started an anti-tuberculosis treatment with rifampicin. However, due to interaction of antiretroviral drugs with rifampicin, the administration of antiretroviral drugs was discontinued. This resulted in a decrease of CD4 counts to 156 cells/ml, while elevated levels of HIV persisted at the end of 1999. Antiretroviral treatment resumed in January 2000, composed of four drugs, namely ddI, stavudine, nevirapine (NVP) and nelfinavir (NFV). As a consequence, CD4 cell counts increased noticeably, and HIV RNA became undetectable. Unfortunately, resistance to therapy was observed during the second semester of 2001, when HIV load reached 1.2 × 10^5 ^copies/ml and CD4 counts decreased to 120 cells/ml. The treatment was then replaced by another including lamivudine associated to AZT, NVP and NFV. No HIV resistance was observed until the end of the follow-up (November 2002). The patient died of renal failure at the end of 2003.

### DNA extraction and PCR assays for DNA sequencing

Three serum samples collected in March 1999, August 2000 and November 2002, were available for HBV DNA analysis. Sera were submitted to DNA extraction by the phenol-chloroform method after treatment with proteinase K, as described previously [17]. HBV pre-S/S genomic region was amplified by PCR using sense primer PS1 (5'-CCATATTCTTGGGAACAAGA-3', nt 2826-2845) and a mix of antisense primers S2 (5'-GGGTTTAAATGTATACCCAAAGA-3', nt 841-819) and S22 (5'-GTATTTAAATGGATACCCACAGA-3', nt 841-819), able to amplify all HBV genotypes. PCR assays were performed under the following conditions: 94°C, 30 s; 52°C, 1 min; 72°C, 2 min; 35 cycles, followed by a final elongation of 7 min at 72°C. Amplification products (10 μL) were loaded on a 2% agarose gel, electrophoresed, stained with ethidium bromide, and visualized under UV light.

### Quantification of HBV DNA

Quantification of HBV DNA was performed by endpoint dilution. DNA samples were diluted to a tenfold series up to 10^-8 ^dilution. Each dilution was submitted to PCR with oligonucleotide pairs PS1–PS2, designed in the pre-S region and C1–C2 (core region), as described previously [4]. Serial dilutions and PCR assays for HBV DNA quantification were performed in triplicate. The sensitivity of the method has been estimated to about 100 copies per PCR reaction [18], equivalent to 10^4 ^HBV genome copies per milliliter of serum. Quantitative results were estimated by dividing 10^4 ^copies/ml by the last positive dilution.

### Molecular cloning and nucleotide sequencing

Pre-S/S PCR products were cloned into pCRII plasmid vector using TA cloning kit (Invitrogen, San Diego, CA). For nucleotide sequencing, recombinant plasmid DNAs were purified by a commercially available kit (Plasmid midi kit, Qiagen, Hilden, Germany). Nucleotide sequences were determined using the Cy5 auto read sequencing kit (Amersham Biosciences, Little Chalfont, UK) with M13 universal and reverse primers, as well as internal, HBV specific primers. For direct sequencing (S region), PCR products were extracted from low melting agarose gels (Qiaquick gel extraction kit, Qiagen). Sequencing reactions with HBV specific primers were done using the thermo sequenase Cy5 dye terminator sequencing kit and analyzed on an ALFexpress automated sequencer (Amersham Biosciences). Independent plus and minus strand sequencing was completed.

### Phylogenetic analysis

Nucleotide sequences were aligned using PILEUP (Wisconsin Sequences Analysis Package GCG, Madison, WI). A phylogenetic tree was generated by neighbour-joining analysis of genetic distances, using the TREECON software package for Windows [19]. HBV sequences available from the GenBank database (accession numbers AY090458, M57663, U55220-U55222, J02201 and X51970) were used for the construction of a phylogenetic tree.

## Results

### Markers of HBV infection

**Figure 1:**
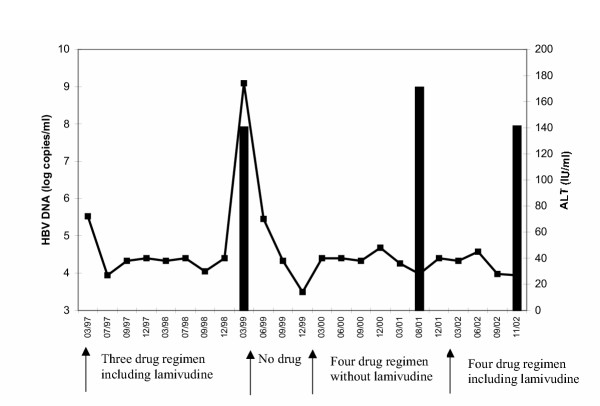
HBV load (vertical bars) and ALT levels (curve) during antiretroviral therapies.

The patient under study developed an acute hepatitis and became anti-HIV positive in 1996, soon after receiving blood transfusions during the initial period of hemodialysis treatment. He became an HBV chronic carrier, with all blood samples collected between 1996 and 2002 being positive for HBsAg and HBeAg. During this period, the patient remained asymptomatic without clinical signs for HBV infection. Aspartate aminotransferase and alkaline phosphatase levels remained normal all during the follow-up. However, an increase of alanine aminotransferase (ALT) levels to 180 IU/L was observed in March 1999 (Figure 1).

HIV treatment started in April 1996. Lamivudine was introduced as part of antiretroviral treatment in November 1996. HBV DNA was detected at high levels (10^8^copies/ml) in the blood sample collected in March 1999, during this first period of lamivudine treatment (Figure 1). In May 1999, lamivudine was discontinued, and the sample analyzed during the period of lamivudine interruption (August 2001) showed the highest (10^9 ^copies/ml) HBV DNA titre mesured in this study. HBV DNA was again detected at high levels (10^8^copies/ml) after reintroduction of lamivudine (November 2002, Figure 1).

### HBV variants

**Figure 2:**
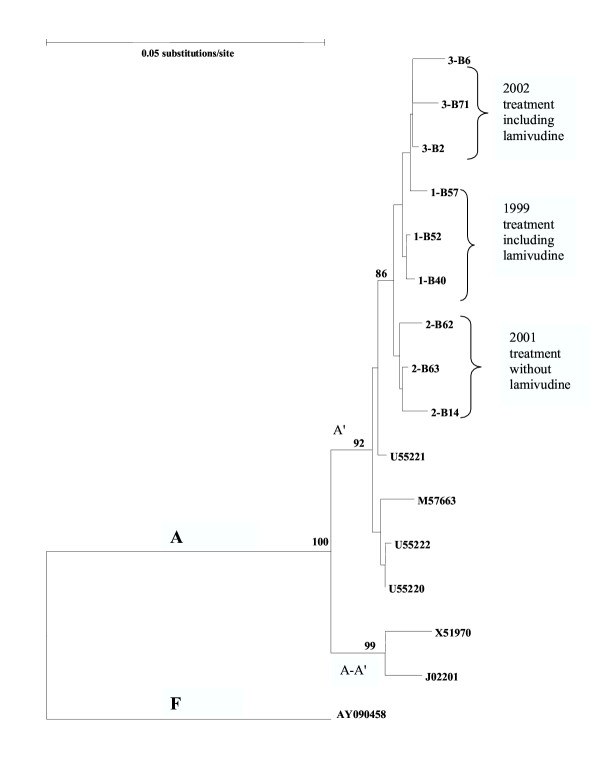
Phylogenetic tree of HBV isolates constructed with the neighbor-joining method, and based on the nucleotide sequences of the entire pre-S/S region (nt positions 2854 to 833). Isolates whose names begin by 1,2,3 were molecular clones from this study derived from samples collected in 1999, 2001, and 2002, respectively. The other isolates are designated by their GenBank accession numbers. Genotype A isolates clustered in two subgroups, designated A-A' (genotype A excluding A') and A' [27]. The sequence AY090458 belongs to genotype F and was used as an outgroup. Numbers at internal nodes indicate percentage of 100 bootstrap replicates that support the branch. Only values > 85% are indicated. The horizontal bar provides a genetic distance.

Nucleotide sequences of HBVs (pre-S/S region) derived from HBsAg/HBeAg positive samples collected in March 1999, August 2001 and November 2002 were determined (three clones each). Phylogenetic analysis showed that all nine clones belonged to genotype A. This genotype has been subdivided into two subgenomic groups, designated A-A' (genotype A excluding A') and A' [20]. Recently, subgroups A-A' and A' were designated respectively as A1 and A2 [21] or Ae and Aa [22]. Isolates belonging to subgroup A' have been first identified in South Africa and circulate in a high proportion among HBV Brazilian isolates [23]. As can be observed on the phylogenetic tree represented in Figure 2, all clones belonged to subgroup A' and were closely related to each other. The six clones obtained during the two periods of lamivudine treatment (1999 and 2002) clustered separately (with a bootstrap value of 86%) from those obtained in 2001, during the period of lamivudine interruption.

Table 1 shows the main amino acid changes observed in the S region of the genome. Two substitutions, H359Y in the polymerase and Y100C in the surface antigen (small S), were observed in all HBV sequences. These two changes were natural variation, characteristics of the HBV strain infecting the patient under study. All three clones derived from the sample collected in 1999, during the first lamivudine treatment, showed two lamivudine resistant mutations (L526M and M550V), also detected in the major viral population by direct sequencing. One clone (1-B57) showed an additional mutation related to drug resistance (V519L) that was not detected by direct sequencing. None of these three mutations associated with lamivudine resistance was observed in sequences derived from the second sample (2001). In contrast, all three clones derived from the third sample (2002) showed the three lamivudine resistant mutations. However, by direct sequencing of PCR products of this last sample, the electropherogram could detect two nucleotides (A and G) at the same sequence position indicating a mixture of V519 and L519 residues. Curiously, all HBV sequences obtained during the period without lamivudine treatment (2001) displayed a unique G473E substitution in the polymerase gene. Stop mutations were also observed in the small S protein, which was truncated at position 182 in clone 2-B14 and at position 216 in clone 2-B62 (Table 1).

Due to overlapping of polymerase and S genes on the HBV genome, mutations at positions 473, 519 and 550 of the polymerase were accompanied by mutations at positions 119, 164 and 195 of the small S protein (Table 1).

## Discussion

Among lamivudine resistant mutations, those in the YMDD motif of the HBV DNA polymerase are the most common. The resistance occurs by replacement of a methionine residue at position 550 by either valine (M550V) or isoleucine (M550I). More rarely, HBV variant presenting M550S replacement may be selected during lamivudine treatment [24] The M550V variant may be accompanied by a mutation (leucine to methionine) at position 526 [25]. In the absence of HIV infection, the mutation at position 550 alone has been found in up to two-thirds of patients with lamivudine-resistant chronic hepatitis B. However, more than 90% of HIV-HBV co-infected patients display the double lamivudine resistant mutation at positions 526 and 550 [12]. Furthermore, the presence of L526M mutation in addition to mutation at position 550 seems to be associated with prolonged lamivudine treatment [26]. The double mutant has been shown to exhibit a 15-fold decrease of the *in vitro *susceptibility to lamivudine [27], since each mutation contributes to the loss of lamivudine sensitivity [28]. It is believed that the L526M mutation, when not accompanied with a mutation in the YMDD motif, does not confer lamivudine resistance [29]. Even so, the L526M mutation has been found alone in patients under lamivudine therapy [30]. Here, both L526M and M550V mutations were detected in all HBV sequences derived from the two blood samples collected during lamivudine treatment (1999 and 2002). Besides these common mutations, a third lamivudine resistant, rare mutation [11,31], namely V519L, was detected in one clone derived from the sample collected in 1999 as well as in all HBV sequences derived from the sample collected in 2002. This was in agreement with recent observations, showing that long lamivudine treatment may result in the predominance of this rare mutant [32]. The triple mutation V519L, L526M, M550V causes the concomitant amino acid substitutions E164D and I195M in the small S protein. It has been shown that this triple mutant has a reduced *in vitro *affinity to anti-HBs antibodies, similar to the hepatitis B vaccine escape mutant G145R [11,31]. The accumulation of mutations in the HBV genome should be monitored in order to guide patient management adequately.

There is a general consensus that the lamivudine resistant single mutants in the YMDD motif (M550V/I) replicate substantially more slowly than the wild type. The addition of the mutation at position 526 may act as a compensatory change that partially restores the replication fitness of the virus [33]. Here, in agreement with this observation, HBV DNA was detected at high levels (about 10^8 ^copies/ml) in the blood sample collected in 1999, in which the double mutant represents the major viral population. Similar high HBV loads were observed in the sample containing the triple mutant (2002). This result is in agreement with a recent study, showing that V519L mutation also enhances viral replication [34].

The HBV population that emerged during the interruption of lamivudine treatment did not show mutations at polymerase positions 519, 526 and 550. However, another substitution, namely G473E, was observed that was associated to G119R substitution in the small S protein. Such a virus might be present as a minor population but not detected during the first lamivudine treatment. Although two out three clones of this population possessed stop codon mutations in S gene, HBV load (10^9 ^copies/ml) was moderately higher during the lamivudine interruption period than that found during lamivudine periods.

HBV chronically infected patients submitted to lamivudine treatment may have acute exacerbation after withdrawal of drug therapy or when lamivudine resistance emerges [8,14-16,26,35-37]. In HIV-HBV co-infected patients, withdrawal of lamivudine may result in severe [8,35,37] or fulminant [37] hepatitis. Factors that trigger severe hepatitis in these cases are not well known. Both viral load and genome variations have been implicated in the pathogenesis of acute exacerbation. Here, the patient under study did not present clinical signs of HBV infection. The absence of acute exacerbation after the emergence of lamivudine resistant variants may be correlated to the absence of significant variations of the viral load. Indeed, HBV DNA was detected at high levels (10^8^–10^9 ^copies/ml) at different periods of antiretroviral treatment with and without lamivudine.

## Conclusions

A rare HBV triple mutant, belonging to genotype A, subgroup A', appeared predominantly in a patient submitted to lamivudine as part of HIV treatment. This type of mutant, previously found in isolates belonging to genotypes A, D and G [15,36], may behave as a vaccine escape mutant. Understanding the circumstances leading to the appearance of such HBV strains may help to guide future therapies in HIV-HBV co-infected patients. A correlation may exist between acute exacerbation of hepatitis B and HBV load in lamivudine treated patients.

## Competing interests

None declared.

## Authors' contributions

EAS and MVS carried out cloning and sequencing of HBV DNA. JA was involved in clinical evaluation of the patient and supervised the antiretroviral treatment. SAG conceived the study, participated in its design and coordination, and wrote the manuscript. All authors read and approved the final manuscript.

## Pre-publication history

The pre-publication history for this paper can be accessed here:



## Figures and Tables

**Table 1:** Amino acid replacements in polymerase and surface antigen (small S)

Year of collect	Lamivudine	HBV sequences	Polymerase residues					S residues					
				
			359	473	519	526	550	100	119	164	182	195	216
1999	Yes	Direct	Y	G	V	**M**	**V**	C	G	E	W	**M**	L
		1-B40	Y	G	V	**M**	**V**	C	G	E	W	**M**	L
		1-B52	Y	G	V	**M**	**V**	C	G	E	W	**M**	L
		1-B57	Y	G	**L**	**M**	**V**	C	G	**D**	W	**M**	L
2001	No	Direct	Y	E	V	L	M	C	R	E	W	I	L
		2-B14	Y	E	V	L	M	C	R	E	*	I	L
		2-B62	Y	E	V	L	M	C	R	E	W	I	*
		2-B63	Y	E	V	L	M	C	R	E	W	I	L
2002	Yes	Direct	Y	G	**L/V**	**M**	**V**	C	G	**D/E**	W	**M**	L
		3-B02	Y	G	**L**	**M**	**V**	C	G	**D**	W	**M**	L
		3-B06	Y	G	**L**	**M**	**V**	C	G	**D**	W	**M**	L
		3-B71	Y	G	**L**	**M**	**V**	C	G	**D**	W	**M**	L
*Consensus of genotype A*			H	G	V	L	M	Y	G	E	W	I	L

Lamivudine resistant mutations in the viral DNA polymerase and their counterparts in the small surface antigen are marked in bold. Asterisks represent stop mutations.
